# Small Semi-Fossorial Herbivores Affect the Allocation of Above- and Below-Ground Plant Biomass in Alpine Meadows

**DOI:** 10.3389/fpls.2022.830856

**Published:** 2022-02-21

**Authors:** Qian Wang, Xiao Pan Pang, Zheng Gang Guo

**Affiliations:** State Key Laboratory of Grassland Agroecosystems, Key Laboratory of Grassland Livestock Industry Innovation, Ministry of Agriculture and Rural Affairs, College of Pastoral Agriculture Science and Technology, Lanzhou University, Lanzhou, China

**Keywords:** alpine meadow, biomass allocation, plateau pika, small semi-fossorial herbivore, soil properties

## Abstract

Small semi-fossorial herbivores can affect plant aboveground biomass (AGB) in grasslands and possibly alter the allocation of AGB and belowground biomass (BGB). In this study, plateau pika (*Ochotona curzoniae*) was used to investigate such effects at three alpine meadow sites on the Eastern Tibetan Plateau, where pairs of disturbed vs. undisturbed plots were randomly selected and sampled. We also explored the relationships between soil properties and BGB/AGB across the plots in the presence and absence of plateau pikas, respectively. We found that BGB and BGB/AGB were 11.40 and 8.20% lower in the presence of plateau pikas than in their absence, respectively. We also found that the BGB/AGB was positively related to soil moisture and soil total nitrogen (STN) in the absence of plateau pikas. In contrast, BGB/AGB was positively related to STN, soil organic carbon (SOC), soil carbon/nitrogen (C/N), and soil total phosphorus in the presence of plateau pikas. These factors indicated plateau pika disturbance increased AGB allocation. The relationship between AGB and BGB of alpine meadow plants to soil variables was also different between sites with and without plateau pika disturbance. In conclusion, small semi-fossorial herbivore disturbance is likely to alter grassland carbon stock and should be well controlled for sustainable conservation and management of alpine meadows on the Tibetan Plateau.

## Introduction

The allocation of aboveground biomass (AGB) and belowground biomass (BGB) is widely used to characterize the carbon pool dynamics of grassland ecosystems (Pausch and Kuzyakov, 2018; Yang et al., 2018), which is closely related to temperature, precipitation, soil properties, and biotic factors (Patty et al., 2010; Gong et al., 2015). However, it is still unclear how herbivores affect plant biomass allocation, especially for alpine meadows of high elevations.

Herbivores are important biotic factors that can affect the allocation of AGB and BGB in grasslands (Frank et al., 2002; Zeng et al., 2015; Sun et al., 2018). Large grazing herbivores have been found to increase (Frank et al., 2002; Zeng et al., 2015; Sun et al., 2018) or decrease (Gao et al., 2008; Gong et al., 2015) the ratio of BGB to AGB. Furthermore, total soil bulk density (SBD) (Abaye et al., 1997) and soil nutrients (Sun et al., 2018) can regulate the allocation of AGB and BGB in the presence of large herbivores, which is mainly related to the fact that grazing grasslands and non-grazing grasslands experience the same temperature and precipitation levels in a given study area (Sun and Wang, 2016). In addition to the large grazing herbivores, numerous small semi-fossorial herbivores are underappreciated. These small semi-fossorial herbivores are key functional components in grassland ecosystems (Davidson et al., 2012; Davies et al., 2019; Smith et al., 2019; Cui et al., 2020) and often create extensive disturbances to vegetation and soil (Davidson et al., 2012; Wilson and Smith, 2015). Previous studies have shown that the presence of small semi-fossorial herbivores can decrease (Poe et al., 2019; Cui et al., 2020), increase (Root-Bernstein and Ebensperger, 2013; Galiano et al., 2014), or have no impact on plant AGB (Pang et al., 2020a). For example, higher population densities of small semi-fossorial herbivores decrease plant BGB (Sun et al., 2015; Liu et al., 2017). Therefore, small semi-fossorial herbivores might modify the allocation of AGB and BGB through their effect on aboveground and belowground interactions (Gao et al., 2008; Deyn, 2017).

Small semi-fossorial herbivores can affect soil properties by burrowing tunnels (Nicod et al., 2020; Andersen et al., 2021), excreting feces and urine (Clark et al., 2016; Cui et al., 2020; Zhang et al., 2020), and redistributing the soil (Davidson et al., 2012; Guo et al., 2012; Pang et al., 2020a). Previous studies have shown that small semi-fossorial herbivores can decrease SBD (Dobson et al., 1998; Wilson and Smith, 2015) and soil moisture (Pang and Guo, 2017) and increase the soil nitrogen concentration (Liu et al., 2013; Yu et al., 2017a; Mallen-Cooper et al., 2019; Cui et al., 2020) and soil organic carbon (SOC) (Clark et al., 2016; Pang et al., 2019) in grasslands. The changes in soil carbon and nitrogen concentrations caused by small semi-fossorial herbivores can affect plant biomass (Yang et al., 2021), which is ultimately determined by the plant community and soil (Patty et al., 2010; Gong et al., 2015; Prommer et al., 2020). Thus, small semi-fossorial herbivores may indirectly affect the allocation of AGB and BGB. However, the effects of disturbance by small semi-fossorial herbivores on the allocation of AGB and BGB in grasslands are not well documented.

Plateau pika (*Ochotona curzoniae*) is a small semi-fossorial herbivore that is philopatric (Dobson et al., 1998), prefers open-vegetation habitats, and lives in family groups (Smith et al., 2019; Wang et al., 2020). Plateau pika families of various sizes often live together within their home range (Fan et al., 1999; Smith et al., 2019) and are distributed patchily in grasslands (Pang et al., 2020b). Open-vegetation habitats free of plateau pikas are considered potentially suitable areas (Li et al., 2021), and the diffusion of plateau pikas through the landscape is a gradual process (Pang et al., 2020b). Disturbance by plateau pikas decreases (Liu et al., 2013) or has no impact on the plant AGB (Pang et al., 2020a) in their home range compared with areas lacking plateau pikas. Disturbance by plateau pikas also decreases SBD (Yu et al., 2017b) and soil moisture (Pang and Guo, 2017; Wang et al., 2018) and increases the soil nitrogen concentration (Qin et al., 2019) and phosphorus concentration (Yu et al., 2017b; Pang et al., 2021). Plateau pikas inhabit alpine meadows varying in dominant plants, topography, soil type, and climate on the Qinghai-Tibetan Plateau (Guo et al., 2012; Smith et al., 2019; Wang et al., 2020). Therefore, data from a single site are insufficient for inferring how disturbance by plateau pikas, coupled with environmental factors, affects the ratio of BGB to AGB (Li et al., 2021). Data from multiple sites with similar environmental conditions with and without pikas are needed to evaluate the general effect of pika disturbance on the allocation of AGB and BGB. In this study, the effect of plateau pikas on the allocation of AGB and BGB in three sites was examined. Specifically, a hypothesis-based structural equation model was used to determine whether and how disturbance by plateau pikas affects the ratio of BGB to AGB. The results of this study enhance our understanding of the effect of small semi-fossorial herbivore disturbance on grassland ecosystem carbon cycling.

## Materials and Methods

### Study Sites

Plots for plant biomass and soil sampling were established at three different sites on the Qinghai-Tibetan Plateau in Gonghe County (35.5°–37.2°N, 99°–101.5°E), Gangcha County (36.9°–38°N, 99.3°–100.6°E), and Luqu County (34°–34.8°N, 101.6°–103°E). The elevations of the three sites were 3,750, 3,265, and 3,505 m; the average annual precipitation was 400, 572.3, and 644 mm; and the average annual temperature was 4.1, 0.89, and 3.16°C, respectively. According to the Chinese Soil Classification System (Gong et al., 2007), the soils of these sites are alpine meadow soils. The alpine meadow at the three sites is divided into warm and cold grazing areas; the cold grazing areas were fenced from mid-April to early October, and these fences were opened for grazing by Tibetan sheep and yak from late October to early April. The dominant plants in the alpine meadows were *Kobresia humilis, K. pygmaea*, and *K. humilis* at the Luqu, Gonghe, and Gangcha sites, respectively. Although there were many small herbivores at the three sites, plateau pikas were the only small semi-fossorial herbivores in the survey areas at each site.

### Experimental Design

Field surveys at each site were conducted in the cold grazing areas of the alpine meadows. A random stratified and paired design was used to establish plots at each site. The plot size was 35 m × 35 m, which was close to the size of the average home range of plateau pikas (1,262.5 m^2^; Fan et al., 1999). First, 10 disturbed plots in which plateau pikas or active burrow entrances were present were selected along the driving route at each site, and the distance between these disturbed plots ranged from 5 to 10 km. Second, a paired undisturbed plot in which plateau pikas and active burrowing entrances were absent was selected for each disturbed plot, and the distance between each disturbed plot and its paired undisturbed plot ranged from 500 to 1,000 m. Thus, the undisturbed plot experienced the same environmental conditions, including temperature, precipitation, and topography, as its paired disturbed plot. The movement of plateau pikas might affect the undisturbed plot if the distance between the paired plots was close; however, the vegetation and topography might differ between paired plots when the distance is large. There was a total of 10 paired plots at each site and 60 plots across the three sites, including 30 disturbed plots and 30 undisturbed plots. Grazing management, including the stocking rate of yaks, was the same for each paired plot in the cold season. In this experimental design, the stocking rate of yaks varied among the 30 paired plots, which permitted the general pattern relating to the effect of plateau pika disturbance on the allocation of AGB and BGB to be determined. In addition, the disturbance intensity by plateau pikas varied among the 30 disturbed plots (which was similar to variation in the degree of degradation of alpine meadows), which permitted the general pattern relating to the effects of plateau pika disturbance on the allocation of AGB and BGB to be determined.

### Field Survey

The field survey was conducted during early August 2017, as this coincides with the peak in the plateau pika population (Davidson et al., 2012; Pang et al., 2019) and thus the level of disturbance. There were five subplots (1 m × 1 m) in each plot that was arranged in a “W” distribution pattern. Although there were many kinds of bare soil patches in alpine meadows, the bare soil patches caused by plateau pikas were easily visible and differed from signs of disturbance caused by other factors (Yu et al., 2017a). Thus, the placement of subplots in the disturbed plots was altered slightly to prevent bare soil patches created by plateau pikas from falling within them. The distance between each subplot was greater than 8 m. In each subplot, plant shoots were harvested by hand at ground level, and soil moisture (SM) was measured using a time-domain reflectometer with five replicates. The plant root system was mainly distributed in the 0–20 cm layer in the alpine meadow, which accounted for approximately 85% of the total BGB (Li et al., 2011b; Xu et al., 2016). Therefore, an auger with a 10 cm diameter was used to collect soil cores in the 0–20 cm layer in the center of each subplot, and this soil core was used to measure the BGB. The soil profile was used to sample 500 g of fresh soil to measure soil pH, SOC, soil available nitrogen (SAN), soil total nitrogen (STN), soil available phosphorus (SAP), and soil total phosphorus (STP). A cutting ring (100 cm^3^, 50.46 mm diameter × 52 mm height) was used to collect soil samples for the measurement of SBD. The plant shoots, soil cores, and soil samples were stored at 4°C.

In the laboratory, plant shoots were dried at 80°C in an oven to a constant weight and weighed to calculate the AGB. Soil cores were placed into 0.45-mm mesh filter gauze bags to collect the BGB by washing. For each soil core, fresh roots were divided into live roots and dead roots by color, consistency, and presence of attached fine roots (Yang et al., 2009; Xu et al., 2016), and the live roots were dried at 80°C to a constant weight to estimate BGB. Soil samples for measuring the SBD were dried at 80°C to a constant weight. The soil samples were air-dried and passed through a 2-mm sieve to remove gravel and roots for measurements of soil pH, SOC, SAN, and SAP and passed through a 0.15-mm sieve to remove gravel and roots for measurements of STN and STP. The soil pH was measured with an acidimeter (PHSJ-6 L, REX, China). SOC was measured using the Walkley (1947) method. Soil nitrate nitrogen (NH_4_^+^-N) and ammonium nitrogen (NO_3_^–^-N) were extracted with potassium chloride (KCl, 2 mol L^–1^), and their concentrations were measured using the flow injection method (FIA star 5000 Analyzer, FOSS, Denmark). STN was measured using the Kjeldahl method (Foss Kjeltec 8400, FOSS, Denmark). STP and SAP were analyzed using inductively coupled plasma spectrometers (UV-2102 PCS, China). SAN was the sum of the NH_4_^+^-N and NO_3_^–^-N concentrations.

The AGB, BGB, BGB/AGB, SM, soil pH, SBD, SOC, STN, SAN, STP, and SAP data for each plot were generated by pooling the values of these variables for each of the five subplots. Soil carbon/nitrogen (C:N) for each plot was the average SOC/STN of the five subplots.

### Statistical Analyses

We first checked the data for normality using the Shapiro-Wilk test. A linear mixed model (LMM) from “lmer” in the lme4 package of R version 4.1.1 (R, Vienna, Austria) was then used to analyze the effects of plateau pika disturbance on the AGB, BGB, and BGB/AGB at each site. In the LMM, AGB, BGB, and BGB/AGB were response variables, the presence/absence of plateau pika disturbance (Dist.) and the three sites (site) were fixed effects, and paired plots within site was a random effect. When site effects were significant, a non-parametric paired *t*-test was used to evaluate the effects of plateau pika disturbance on the AGB, BGB, and BGB/AGB at each site.

Pearson’s rank correlations were used to select the explanatory variables and subordinate explanatory variables by testing for the significance of relationships of BGB/AGB with soil physical properties (SM, SBD, and soil pH) and soil chemical properties (SOC, SAN, STN, STP, SAP, and soil C:N). The main explanatory variables were soil variables that were significantly related to BGB/AGB (*p* < 0.05); soil variables not showing significant relationships to BGB/AGB were common explanatory variables. Subordinate explanatory variables were common explanatory variables that were significantly related to each of the main explanatory variables (*p* < 0.05).

Generalized additive models (GAMs) in the “mgcv” package in R were used to analyze the effect of each main explanatory variable on the BGB/AGB in the presence and absence of plateau pika (Wood et al., 2017). Sites differing in elevation and precipitation were included as covariates because these variables were suspected *a priori* to affect the ratio of BGB to AGB. Each main explanatory variable was included in the model as smoothers. GAMs were selected based on the *p*-value (*p* < 0.05) and Akaike information criterion (AIC) (δAICc < 2).

**FIGURE 1:**
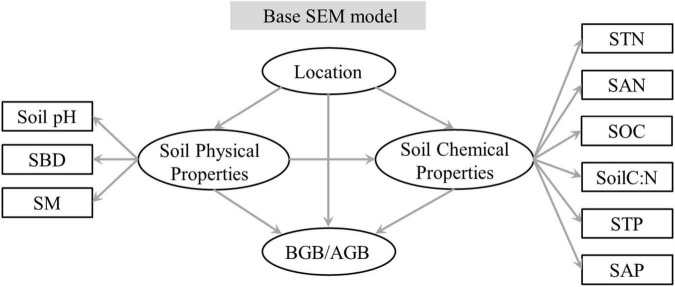
Graphical representation of the hypothesis-based structural equation model of site and soil properties on BGB/AGB. Significant variables were included to predict BGB/AGB. AGB, aboveground biomass; BGB, belowground biomass.

To quantify the integrated effect of the main explanatory variables and subordinate explanatory variables on BGB/AGB, a structural equation model (SEM) in the “lavaan” package in R was used to identify the pathway and direct or indirect effect of each main and subordinate explanatory variable. As soil properties are mutual interaction between AGB and BGB, BGB/AGB was used to construct an SEM. A hypothesis-oriented pathway was used to construct a base SEM (Figure 1). The three sites represented a single variable group; the main and subordinate explanatory variables from the Pearson’s rank correlations were then classified into soil physical and chemical property variable groups; and the SEM analysis and a path model were used to examine the direct and indirect effects of the three variable groups on BGB/AGB. The chi-square (χ^2^) statistic, whole model *p*-value, goodness-of-fit index (GFI), and root-mean-square error of approximation (RMSEA) were used to optimize the base SEM and improve model fit. This improved model had low χ^2^-values, low RMSEA values (< 0.08), high GFI values (> 0.96), and low *p*-values (> 0.05), and the differences between this model and observed values were the lowest.

## Results

### Aboveground Biomass, Belowground Biomass, and Belowground Biomass/Aboveground Biomass

**FIGURE 2:**
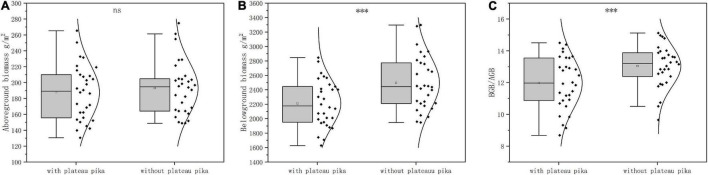
The **(A)** plant AGB, **(B)** BGB, and **(C)** ratio of BGB and AGB in the presence and absence of plateau pikas across three sites. The statistics were from the linear mixed model, in which the three sites (site) and disturbance by plateau pikas (Dist.) were fixed effects, and paired plots nested within the site were a random effect. The quadrilateral points in the box represent the average of variables in the presence and absence of plateau pikas. *Significant differences at *p* < 0.05, ^**^p < 0.01, ^***^p < 0.001, ns *p* > 0.05.

**FIGURE 3:**
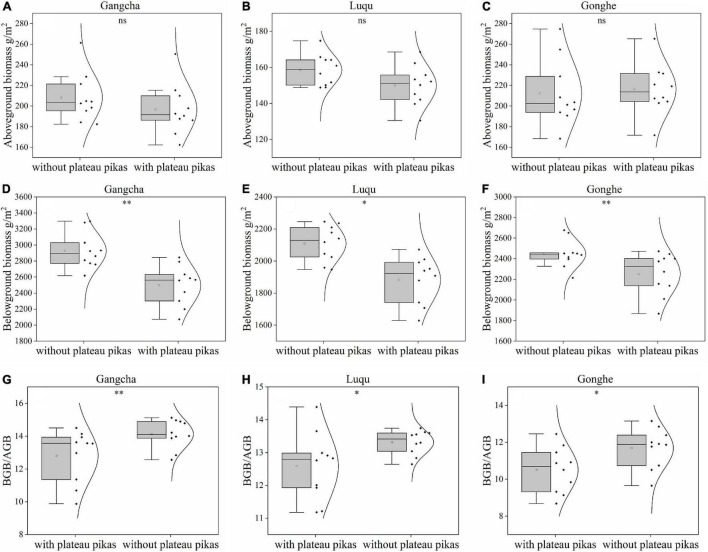
AGB, BGB, and ratio of BGB and AGB in the presence and absence of disturbance by plateau pikas at Gangcha (**A**: AGB, **D**: BGB, **G**: BGB/AGB), Gonghe (**B**: AGB, **E**: BGB, **H**: BGB/AGB), and Luqu (**C**: AGB, **F**: BGB, **I**: BGB/AGB). *Significant differences at *p* < 0.05, ^**^p < 0.01, ^***^p < 0.001, ns *p* > 0.05.

When the data from the three sites were analyzed together, the plant BGB and BGB/AGB were 11.40 and 8.20% lower in the presence of plateau pikas than in their absence, respectively; plant AGB did not significantly differ among plots with and without plateau pikas (Figure 2). When data from individual sites were analyzed separately, the responses of AGB, BGB, and BGB/AGB to disturbance by plateau pikas were consistent at each site (Figure 3).

### Pearson’s Rank Correlations Between Soil Properties and Belowground Biomass/Aboveground Biomass and Generalized Additive Model Analysis

**FIGURE 4:**
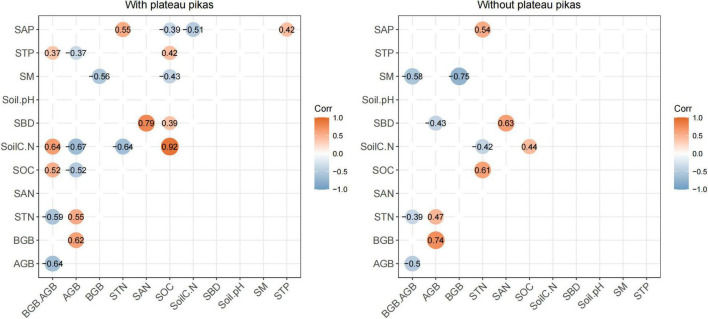
The relationships of BGB/AGB with environmental factors in areas with and without plateau pikas. Orange indicates a positive correlation at *p* < 0.01, and blue indicates a negative correlation at *p* < 0.01. STN, soil total nitrogen; SAN, soil available nitrogen; SOC, soil organic carbon; soil C:N, soil carbon/soil nitrogen; STP, soil total phosphorus; SAP, soil available phosphorus; SM, soil moisture; SBD, soil bulk density.

Pearson’s rank correlations indicated that BGB/AGB was significantly correlated with STN (*R*^2^ = 0.63, *p* < 0.05), SOC (*R*^2^ = 0.44, *p* < 0.05), soil C:N (*R*^2^ = 0.55, *p* < 0.05), and STP (*R*^2^ = 0.40, *p* < 0.05) in the presence of plateau pikas and with SM (*R*^2^ = 0.59, *p* < 0.05) and STN (*R*^2^ = 0.40, *p* < 0.05) in the absence of plateau pikas (Figure 4), indicating that the disturbance caused by plateau pikas altered the relationships of BGB/AGB with SM and STN. Thus, SM, STN, SOC, soil C:N, and STP were the main explanatory variables.

Soil organic carbon was the only main explanatory variable significantly correlated with SBD in the presence of plateau pikas (*R*^2^ = 0.39, *p* < 0.05); no other soil property variables were significantly related to each of the main explanatory variables in the absence of plateau pikas. These results indicated that SBD was a subordinate explanatory variable.

**FIGURE 5:**
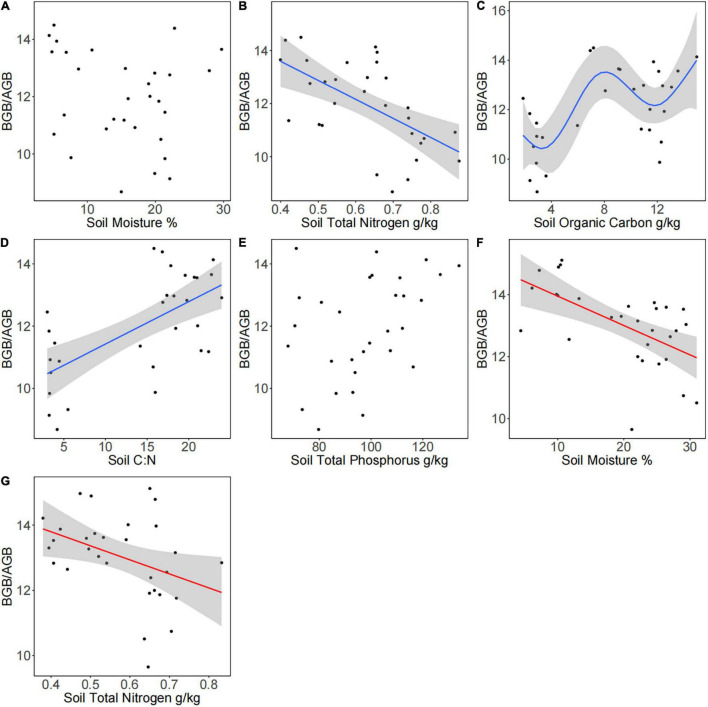
Relationships between environmental factors and the ratio of BGB to AGB in the presence and absence of plateau pikas in alpine meadows. The effects of **(A)** soil moisture, **(B)** STN, **(C)** SOC, **(D)** soil C:N, and **(E)** STP on the BGB/AGB in the presence of plateau pikas. The effects of **(F)** SM and **(G)** STN on BGB/AGB in the absence of plateau pikas. Ten replicates within each of the three sites in the presence (*n* = 30) and absence (*n* = 30) of plateau pikas are presented. The best-fit GAM had a significant *p*-value (*p* < 0.5), the lowest Akaike information criterion (AIC), the lowest generalized cross-validation (GCV), and the highest *r*-squared values, and site was included as a random factor.

According to the GAM analysis, there was no significant linear or non-linear relationship of BGB/AGB with SM and STP; however, there was a negative linear relationship of BGB/AGB with SM, soil C:N, and STN in the presence of plateau pikas. BGB/AGB increased in a fluctuating manner as SOC increased from approximately 8 to 12 g/kg in the presence of plateau pikas. There was a negative linear relationship of BGB/AGB with SM and STN in the absence of plateau pikas (Figure 5 and Table 1).

**TABLE 1:** Generalized additive models of the relationships of BGB/AGB with plant community biomass and soil properties in the presence and absence of plateau pika.

	df	GCV	F	*p*	*R* ^2^
**Plateau pikas present**					
Soil moisture	1.848	2.774	2.311	0.109	0.151
Soil total nitrogen	1	1.980	14.570	0.000	0.319
Soil organic carbon	5.487	1.622	5.953	0.000	0.515
Soil C:N	1	1.888	19.330	0.000	0.387
Soil total phosphorus	2.555	2.483	2.994	0.070	0.178
**Plateau pikas absent**					
Soil moisture	4.587	1.278	4.194	0.000	0.375
Soil total nitrogen	1	1.613	4.979	0.001	0.121

*Df = 1 denotes the linear relationship between two variables. AGB, aboveground biomass; BGB, belowground biomass.*

### Relationships Between Belowground Biomass/Aboveground Biomass and Explanatory Variables According to the Structural Equation Model

**FIGURE 6:**
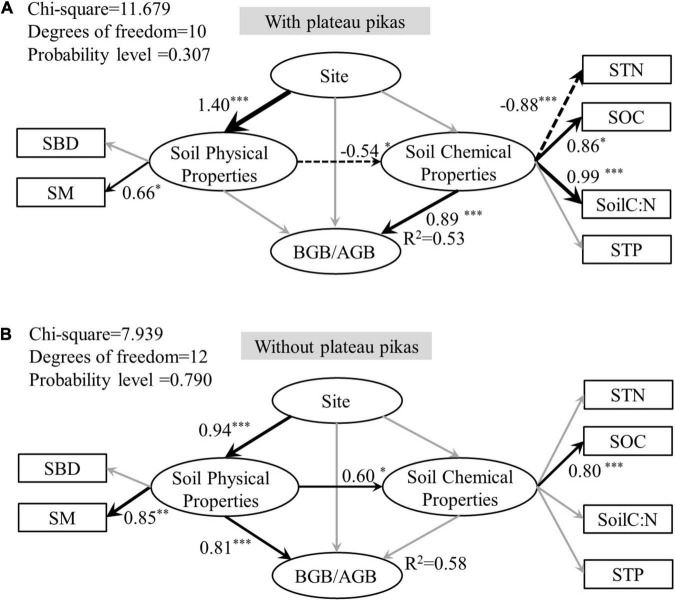
Structural equation model (SEM) exploring the direct and indirect effects and standardized total effects of site, soil physical properties (SBD, SM), and soil chemical properties (STN, SOC, soil C:N, STP) on BGB/AGB in areas with **(A)** or without **(B)** plateau pikas. The black arrows indicate that the effect is significant, and gray arrows indicate insignificant effects. Asterisks next to path coefficients indicate *p-*values. *p < 0.05, ^**^p < 0.01, ^***^p < 0.001. Solid lines and dotted line arrows indicate positive and negative significant relationships, respectively, and arrow thickness indicates the strength of the relationships. STN, soil total nitrogen; SOC, soil organic carbon; Soil C:N, soil carbon/soil nitrogen; SM, soil moisture; SBD, soil bulk density; AGB, above-ground biomass; BGB, below-ground biomass; BGB/AGB, below-ground biomass/above-ground biomass.

The results of the SEM analysis explained 53 and 58% (*R*^2^ = 0.53 and 0.58) of the variance in BGB/AGB in the presence and absence of plateau pikas, respectively. The SEM analysis showed that BGB/AGB at each site was affected through different pathways in the presence and absence of plateau pikas (Figure 6).

## Discussion

Large grazing herbivores have been found to affect the allocation of AGB and BGB in shrub-steppes (Bagchi and Ritchie, 2010), semiarid grasslands (Patty et al., 2010), and alpine grasslands (Sun and Wang, 2016) and contribute to carbon cycling in grassland ecosystems (Hermans et al., 2006; Bardgett and Wardle, 2010; Pausch and Kuzyakov, 2018; Yang et al., 2018). In this study, multiple sites were used to examine the effect of plateau pikas on the allocation of AGB and BGB in alpine meadow ecosystems.

The results of our study revealed that disturbance by plateau pikas had no impact on AGB, but it decreased BGB and BGB/AGB, which indicates that plateau pikas can affect the allocation of AGB and BGB. This is consistent with the result of some previous studies (Pang and Guo, 2017; Pang et al., 2020a) but inconsistent with the results of others (Liu et al., 2013). This variation in the effect of plateau pikas on AGB among studies likely stems from differences in the placement of sampling plots. Paired plots were used in this study, and each set of paired plots (disturbed plot and undisturbed plot) was placed in the same alpine meadow type, similar to studies carried out by Pang and Guo (2017) and Pang et al. (2020a). In contrast, Liu et al. (2013) placed plots with or without plateau pikas in areas that differed in dominant plants; thus, low plant AGB in plots with plateau pikas compared with plots without plateau pikas could stem from differences in plateau pika presence of vegetation.

Although the consumption of plants by plateau pikas can decrease AGB (Smith et al., 2019; Pang et al., 2020a), there are three ways that plateau pika disturbance can increase AGB. First, herbivory by plateau pikas can lead to compensatory plant growth (McNaughton, 1983). Second, plateau pikas clip older and taller plant tissues (Liu et al., 2009) to increase light availability for shorter plants (Pang et al., 2020a; Zhang et al., 2020). Third, higher concentrations of SAN in the presence of plateau pikas (Yu et al., 2017a) are beneficial for the accumulation of nitrogen in leaves and can enhance the allocation of photosynthate to the aboveground parts of plants. The results of the statistical analyses indicate that plateau pika disturbances appear to have a zero-net effect on plant AGB; however, this stems from the fact that the increases in AGB *via* the aforementioned three consequences of plateau pika disturbance are offset by the decrease in AGB associated with plant consumption.

Plateau pika disturbance contributes to a decrease in BGB in three ways. First, plateau pika disturbance increases the heterogeneity of the soil (Pang et al., 2019; Zhang et al., 2020), and this enhancement of the habitat promotes the conversion of live fine roots to dead fine roots (Bardgett and Wardle, 2010). Second, plants require fewer roots to acquire nutrients because nutrient concentrations are higher in the presence of plateau pikas (Bagchi and Ritchie, 2010; Kiaer et al., 2013; Yu et al., 2017a; Maskova and Herben, 2018; Cleland et al., 2019; Qi et al., 2019). Third, the higher soil nitrogen concentration in the presence of plateau pikas (Yu et al., 2017a) can increase the mortality of roots (Bai et al., 2008; Li et al., 2011a). The response of BGB/AGB to plateau pika disturbance depends on changes in both AGB and BGB. Low BGB/AGB in the presence of plateau pikas indicates that plateau pika disturbance permits plants to allocate more biomass to the aboveground parts.

The main soil factors affecting BGB/AGB differed in the presence and absence of plateau pikas. The presence of plateau pikas increased the net effect of soil chemical properties on BGB/AGB but decreased the net effect of soil physical properties on BGB/AGB. BGB/AGB is closely related to SM and STN in the absence of plateau pikas; however, in the presence of plateau pikas, BGB/AGB is closely related to STN, SOC, soil C:N, and STP, suggesting that disturbance by plateau pikas can alter the main soil physical and chemical factors that control BGB/AGB. There are three likely causes for the differences in the soil properties most closely related to BGB/AGB in the presence and absence of plateau pikas. First, compared with alpine meadow microhabitats with small semi-fossorial herbivores, alpine meadow microhabitats without small semi-fossorial herbivores are relatively stable (Bagchi and Ritchie, 2010), and the soil nutrients and light sources at these sites are relatively homogeneous (Pang et al., 2020a). In a stable, homogeneous microhabitat, SM changes more readily than soil nutrients in the short term; consequently, SM is one of the main factors affecting the allocation of AGB and BGB in the absence of plateau pikas. Second, plateau pika disturbance increases heterogeneity in the amount of fertilizer in alpine meadows (Yu et al., 2017b), and the uneven distribution of soil nutrients in the presence of plateau pikas enables plants to compete for soil nutrients to maintain their growth. In addition, the higher SOC in the presence of plateau pikas is closely related to soil nitrogen. Thus, STN, SOC, soil C:N, and STP were the main factors affecting the allocation of AGB and BGB in the presence of plateau pikas. Third, selective consumption and clipping by plateau pikas (Smith et al., 2019; Pang et al., 2020a; Wang et al., 2020) can stimulate the compensatory growth of the aboveground parts of plants (Wang et al., 2018; Pang et al., 2020a), which enables plant roots to absorb more soil nutrients to maintain the growth of the entire plant. This increases the importance of soil nutrients and carbon in the presence of plateau pikas and explains why SOC, soil C:N, and STP were the variables most closely related to the allocation of AGB and BGB in the presence of plateau pikas.

## Conclusion

The effects of plateau pikas on the allocation of AGB and BGB were examined across three sites ranging in elevation from 3,265 to 3,750 m and ranging in average annual precipitation from 250 to 633 mm. BGB and BGB/AGB were lower in the presence of plateau pikas than in their absence, and AGB did not vary in the presence and absence of plateau pikas. The main factors affecting the allocation of AGB and BGB might shift from SM and STN in the absence of plateau pikas to SOC, STN, soil C:N, and STP in the presence of plateau pikas. These results reveal the general effects of small semi-fossorial herbivores on the allocation of AGB and BGB and provide new insight into the relationships between small semi-fossorial herbivores and the carbon stock in grassland ecosystems.

## Data Availability Statement

The original contributions presented in the study are included in the article/Supplementary Material, further inquiries can be directed to the corresponding author/s.

## Author Contributions

QW and ZG designed the experiments, analyzed the data, and wrote the manuscript. QW and XP performed the experiments. All authors read and approved the manuscript.

## Conflict of Interest

The authors declare that the research was conducted in the absence of any commercial or financial relationships that could be construed as a potential conflict of interest.

## Publisher’s Note

All claims expressed in this article are solely those of the authors and do not necessarily represent those of their affiliated organizations, or those of the publisher, the editors and the reviewers. Any product that may be evaluated in this article, or claim that may be made by its manufacturer, is not guaranteed or endorsed by the publisher.
