# Supplementary material for: Small Semi-Fossorial Herbivores Affect the Allocation of Above- and Below-Ground Plant Biomass in Alpine Meadows
Source: Front Plant Sci. 2022 Feb 21;13:830856. doi: 10.3389/fpls.2022.830856 (PMC8898946; doi:10.3389/fpls.2022.830856)
Supplement: Supplementary file 1 [file Data_Sheet_1.docx]

TABLE S1 The above-ground biomass and below-ground biomass in relation to the presence/absence of plateau pikas (disturbances) in alpine meadows of three study sites based on linear mixed models

| Response variable | Linear mixed models (Paired design as a random factor) | | | |
| --- | --- | --- | --- | --- |
|  | Dist | | Site | |
|  | Chisq | *p* value | Chisq | *p* value |
| Above-ground biomass | 1.125 | 0.289 | 75.694 | 0.000 |
| Below-ground biomass | 36.379 | 0.000 | 153.475 | 0.000 |

Above-ground biomass and below-ground biomass acted as response variables, while the predictors were the presence/absence of plateau pikas (Dist.), the three study sites (Site). The factor of the paired design of the presence and absence of plateau pikas acted as a random variable.

TABLE S2. The ratio of below-ground biomass and above-ground biomass in relation to the presence/absence of plateau pikas (disturbances) in alpine meadows of three study sites based on linear mixed models

| Response variable | Linear mixed models (Paired design as a random factor) | | | |
| --- | --- | --- | --- | --- |
|  | Dist | | Site | |
|  | F | *p* value | F | *p* value |
| BGB/AGB | 17.278 | 0.000 | 52.055 | 0.000 |

Below-ground biomass/above-ground biomass acted as response variables, while the predictors were the presence/absence of plateau pikas (Dist.), the three study sites (Site). The factor of the paired design of the presence and absence of plateau pikas acted as a random variable.
